# L'asthme allergique au centre tunisien

**DOI:** 10.11604/pamj.2015.20.133.5642

**Published:** 2015-02-16

**Authors:** Samah Joobeur, Saousen Cheikh Mhamed, Ahmed Ben Saad, Hathami Mribah, Asma Dekhil, Naceur Rouatbi, Ali El Kamel

**Affiliations:** 1 Service de Pneumologie et d'Allergologie, Hôpital Universitaire Fattouma Bourguiba, Monastir, Tunisie

**Keywords:** Asthme allergique, tests cutanés allergologiques, allergènes, contrôle, allergic asthma, Allergy skin tests, allergens, control

## Abstract

L'asthme allergique pose un réel problème de santé publique vu sa prévalence et son coût de prise en charge élevés. Etudier le profil clinique, fonctionnel respiratoire, allergologique, thérapeutique et évolutif de l'asthme allergique dans une région du centre tunisien. Etude rétrospective portant sur 1132 dossiers de patients porteurs d'asthme allergique suivis dans le service de pneumologie et d'allergologie à l'hôpital de Monastir (Tunisie). L’âge moyen est de 27 ± 12,5 ans. 61,1% des patients sont âgés entre 16 et 39 ans. Une prédominance féminine est notée (56,7%). L'identification de l'allergène en cause s'est basée essentiellement sur les tests cutanés allergologiques (99,4%). Les principaux pneumallergènes identifiés sont les acariens (91,2%), suivis par les pollens (22,8%) et les phanères des animaux (12%). La classification selon la sévérité a conclu à un asthme intermittent à persistant léger chez 87.1% de nos patients. Le traitement s'est basé essentiellement sur la corticothérapie inhalée (67,6%). L'asthme dans notre étude a été jugé contrôlé dans 68,3% des cas, partiellement contrôlé dans 24,8% et non contrôlé dans 6,9% des cas. L'asthme allergique est une affection répandue qui touche essentiellement le sujet jeune en pleine activité. Une prise en charge adéquate permet de contrôler la maladie et de réduire ses répercussions sur le patient et la collectivité.

## Introduction

L'asthme allergique pose un réel problème de santé publique vu sa prévalence et son coût de prise en charge élevés. Etudier le profil clinique, fonctionnel respiratoire, allergologique, thérapeutique et évolutif de l'asthme allergique dans une région du centre tunisien. Etude rétrospective portant sur 1132 dossiers de patients porteurs d'asthme allergique suivis dans le service de pneumologie et d'allergologie à l'hôpital de Monastir (Tunisie). L’âge moyen est de 27 ± 12,5 ans. 61,1% des patients sont âgés entre 16 et 39 ans. Une prédominance féminine est notée (56,7%). L'identification de l'allergène en cause s'est basée essentiellement sur les tests cutanés allergologiques (99,4%). Les principaux pneumallergènes identifiés sont les acariens (91,2%), suivis par les pollens (22,8%) et les phanères des animaux (12%). La classification selon la sévérité a conclu à un asthme intermittent à persistant léger chez 87.1% de nos patients. Le traitement s'est basé essentiellement sur la corticothérapie inhalée (67,6%). L'asthme dans notre étude a été jugé contrôlé dans 68,3% des cas, partiellement contrôlé dans 24,8% et non contrôlé dans 6,9% des cas. L'asthme allergique est une affection répandue qui touche essentiellement le sujet jeune en pleine activité. Une prise en charge adéquate permet de contrôler la maladie et de réduire ses répercussions sur le patient et la collectivité.

## Méthodes

Il s'agit d'une étude rétrospective portant sur 1132 dossiers de patients porteurs d'asthme allergique hospitalisés dans le service de pneumologie et d'allergologie et/ou suivis à la consultation au Centre Hospitalo-universitaire de Monastir durant la période allant de janvier 1997 à novembre 2011. Le diagnostic de l'asthme est basé sur une symptomatologie clinique et/ou fonctionnelle respiratoire compatible. Le diagnostic de l'allergie est basé sur les critères suivants: la présence d'un test cutané allergologique positif (TCA) à un ou plusieurs allergènes. Les allergènes testés comprenaient des extraits d'acariens (Dermatophagoïdes pteronyssinus et Dermatophagoïdes farinea), des extraits de pollens (olivier, 5 graminées, 4 céréales, herbacés et cyprès), des extraits de phanères d'animaux domestiques (chat et chien), des extraits de moisissures (alternaria, aspergillus) et des allergènes de la blatte germanique. Et/ou Un dosage sérique des IgE spécifiques positif par la technique RAST. Les données collectées ont été saisies et analysées sur SPSS 18.0. L’étude statistique a consisté en une analyse descriptive de toutes les données continues et discontinues. Les résultats sont exprimés en moyenne ± déviation standard.

## Résultats

### Caractéristiques cliniques et fonctionnelles respiratoires

Au moment du diagnostic, l’âge de nos patients a varié de 4 à 71 ans, avec une moyenne de 27 ± 12,5 ans. Les enfants (entre 4 et 15 ans) ont représenté 18,4% de notre population. Les adolescents et adultes jeunes (16 à 39 ans) ont représenté 60,1% de notre population. La répartition selon le sexe a montré une légère prédominance du sexe féminin qui a représenté 56,7% de notre population. La répartition en fonction de l’âge et du sexe a montré une nette prédominance masculine jusqu’à l’âge de 18 ans (62% versus 38%). Le sexe ratio dans cette tranche d’âge était de 1,63. Après l’âge de 18 ans, on a constaté une inversion de cette répartition en faveur de la population féminine qui est devenue encore plus nette après 40 ans avec un sexe ratio de 0,45. Des antécédents familiaux d'atopie ont été retrouvés dans 374 cas. Le terrain atopique le plus souvent retrouvé chez les parents était l'asthme (présent dans 297 cas soit 26,2%). Les manifestations allergiques associées à l'asthme ont été retrouvées dans 1011 cas (89,3%). Elles étaient dominées par la rhino-sinusite et la conjonctivite (respectivement présentes dans 78,1% et 35,9% des cas). L’âge de début de la symptomatologie d'asthme a varié de 1 an à 66 ans avec une moyenne de 22,6 ± 11,9 ans. Dans la moitié des cas, l'asthme a débuté entre l’âge de 10 et 27 ans. 108 patients (9,5%) avaient un asthme tardif ayant débuté à un âge supérieur ou égal à 40 ans. La dyspnée paroxystique était le symptôme majeur, présent dans 1029 cas (90,9%) avec un caractère sifflant dans 848 cas (74,9%). La toux sèche a été notée dans 270 cas (23,9%) alors que la sensation d'oppression thoracique n’était présente que dans 104 cas (9,2%). La survenue essentiellement nocturne de ces symptômes a été notée dans 43,1% des cas (Tableau 1).

**Tableau 1:** Caractéristiques cliniques des patients

Caractéristiques	Moyenne ± DS	Nombre (Pourcentage)
Age moyen	27 ± 12,5 ans	
Sexe féminin		642 (56,7%)
Tabagisme		104 (9,2%)
Antécédents familiaux d'atopie		374 (33%)
Antécédents personnels d'atopie		1011 (89,3%)
-Rhinite allergique		884 (78,1%)
-Conjonctivite allergique		408 (35,9%)
Age de début des symptômes	22,6 ± 11,9 ans	
Symptomatologie clinique :		
- dyspnée sifflante		848 (74,9%)
- toux sèche		270 (23,9%)
- oppression thoracique		104 (9,2%)

L'exploration ventilatoire a comporté une spirométrie simple complétée, en cas de trouble ventilatoire obstructif (TVO), par un test de réversibilité aux broncho-dilatateurs. La spirometrie a été pratiquée chez 1024 patients. Pour les autres 108 patients, cet examen était impossible à réaliser pour diverses raisons: soit qu'il s'agissait de petits enfants soit par défaut de coopération de certains patients. Cette exploration ventilatoire a permis d'objectiver la présence d'un trouble ventilatoire obstructif (TVO) chez 270 patients (26,4%). Chez le reste des patients, la spirométrie a objectivé une baisse des débits périphériques dans 282 des cas. Le test de réversibilité aux broncho-dilatateurs (BD) a été réalisé chez 236 patients porteurs de TVO. Le test était positif chez 130 patients (55,1%). 35 patients ont nécessité le recours au test de provocation bronchique non spécifique à la métacholine pour confirmer le diagnostic d'asthme.

### Bilan allergologique et allergènes

La confirmation de l'allergène en cause a été réalisée par les tests cutanés allergologiques dans 1125 cas (99.4%) et par le dosage des lgE spécifiques dans 21 cas dont 20 étaient des enfants. Chez 7 patients le diagnostic d'allergène était porté uniquement par le dosage des IgE spécifiques alors que chez les 14 autres patients les résultats des IgE spécifiques étaient concordants avec ceux des TCA. L'analyse des principaux allergènes de l'asthme a montré une nette prédominance des acariens (1032 cas: 91,2%). Les pollens viennent en deuxième position (258 cas: 22,8%), suivis par les phanères des animaux (136 cas: 12%), les blattes (60 cas: 4,9%), les moisissures (42 cas: 3,7%) (Tableau 2). La répartition selon le nombre d'allergènes a montré une prédominance de la monosensibilisation, observée chez 862 patients (76,6%).

**Tableau 2:** Répartition des allergènes

Allergènes		Nombre	Pourcentage
**Acariens**	DPT	1029	90,9%
	DF	945	83,5%
**Pollens**	Graminées	34	3,0%
	Céréales	108	9,5%
	Oliviers	69	6,1%
	Herbacées	29	2,6%
	Cyprès	18	1,6%
**Phanères des animaux**	Chat	86	7,6%
	Chien	50	4,4%

### Classification de l'asthme

En se basant sur les données cliniques (fréquence des symptômes, sévérité des crises, fréquence des exacerbations et nécessité d'hospitalisation), fonctionnelles (sévérité du TVO) et thérapeutiques (traitement nécessaire) de chaque patient, nous avons pu classer nos asthmatiques selon la sévérité de leur maladie. Il en ressort que l'asthme était intermittent dans 36,4%, persistant léger dans 50,7%, persistant modéré dans 11% et persistant sévère dans 1,9% des cas. Le suivi de ces asthmatiques a permis d'apprécier aussi le contrôle de la maladie en se basant sur les symptômes des patients lors des dernières consultations (fréquence des symptômes, fréquence des exacerbations, leurs besoins en BD et les données de l'EFR dans certains cas). Ainsi, dans notre étude, l'asthme a été jugé contrôlé dans 68,3% des cas, partiellement contrôlé dans 24,8% et non contrôlé dans 6,9% des cas.

### Traitement et évolution

Les β2 adrénergiques inhalés de courte durée d'action ont été prescrits à tous nos patients. La corticothérapie inhalée (CI) a été prescrite dans 765 cas (67,6%) à des doses variant de 200 à 2000 µg/jour d’équivalent béclométhasone. Les β2 adrénergiques inhalés de longue durée d'action sont relativement chères et ne sont toujours pas encore inclus dans la nomenclature hospitalière dans notre pays ainsi que l'association corticothérapie inhalée - β2 adrénergique longue action et c'est seulement depuis quelques années que la caisse nationale d'assurance maladie (CNAM) a pris en charge de façon intégrale l'asthme et ainsi la prescription de ces produits est de plus en plus aisée. Les β2 adrénergiques inhalés de longue durée d'action ont été prescrits chez 50 patients. Une alternative aux β2 adrénergiques de longue durée d'action était la théophylline retard qui a été prescrite dans 204 cas (18%). Les β2 adrénergiques per os ont été prescrits dans 101 cas (8,9%). Six patients (0,5%) étaient sous corticothérapie per os pour asthme mal contrôlé sous corticothérapie inhalée et bronchodilatateurs longue action. Pour le traitement étiologique, des conseils d’éviction des allergènes incriminés (surtout en cas d'allergie aux animaux domestiques), ont été réalisés chez tous nos patients. L'immunothérapie spécifique (ITS) a été réalisée chez 141 patients (12,5%) qui étaient monosensibilisés dans 83,6% des cas. Il s'agissait essentiellement de désensibilisation aux acariens dans 98,5% des cas puis aux pollens. Les sujets qui ont eu une ITS avaient un asthme intermittent à persistant léger dans 85,8% des cas. Cette ITS a été réalisée chez des asthmatiques ayant une rhinite allergique associée dans 85,7% des cas. Une amélioration clinique a été notée chez 108 patients. Le suivi de nos patients nous a permis, en plus de l’évaluation du niveau de contrôle de l'asthme, d'identifier les autres aspects évolutifs de la maladie. 46 patients ont été hospitalisés au service de pneumologie pour des exacerbations aigues de l'asthme. 15 patients ont nécessité l'hospitalisation en unité de soins intensifs pour asthme aigu grave avec recours à la ventilation mécanique dans 7 cas. Une rémission, définie par l'absence de symptomatologie d'asthme alors que le patient ne recevait plus aucun traitement durant deux années ou plus, a été notée dans 44 cas (3,9%). 55% des patients en rémission étaient des enfants et des adolescents (entre 4 et 18 ans).

## Discussion

L'asthme constitue un important problème mondial de santé publique. Sa prévalence est en augmentation dans la majorité des pays, plus particulièrement parmi les enfants [1]. L'asthme est une affection polymorphe. Ses étiologies sont multiples et variées mais dominées dans la totalité des séries par l'allergie [2–4]. La moyenne d’âge de nos patients au moment du diagnostic était 27 ± 12,5 ans avec une majorité (76,8%) d'enfants et d'adultes jeunes. En effet, chez l'enfant, l'asthme est d'origine allergique dans plus de 90% des cas même s'il n'est pas toujours facile de mettre clairement en évidence l'allergène impliqué [4]. Cependant, même si sa fréquence est décroissante en fonction de l’âge, la composante atopique chez les asthmatiques âgés ne doit pas être méconnue [5]. De point de vue sexe, des facteurs génétiques et hormonaux semblent être impliqués dans la répartition de l'asthme selon le sexe [6, 7]. Dans notre série, on a noté une prédominance masculine avant l’âge de 18 ans. Cette prédominance masculine s'efface à l’âge adulte et s'inverse particulièrement après la quarantaine. Cette évolution a été rapportée aussi par Yao T-C [8] et Blanc FX [9]. L'asthme allergique est une maladie à caractère familial. Un grand nombre de gènes ont été identifiés qui prédisposent à la maladie allergique et qui peuvent se transmettre à la descendance [10]. L'asthme parait la principale atopie familiale observée dans la majorité des études [4, 11]. Chez nos patients, l'atopie familiale a été identifiée dans 33% des cas. L'atopie personnelle constitue aussi un facteur de risque d'asthme [12]. La rhinite allergique est la principale manifestation allergique associée à l'asthme allergique [13, 14]. Chez nos patients nous avons retrouvé une rhinite allergique dans 89,3% des cas. La rhinite précède souvent la maladie asthmatique et semble pour certains auteurs précipiter l'asthme [15]. Sa prise en charge précoce et adaptée pourrait prévenir l'apparition de l'asthme [16]. Dans le bilan de l'asthme l'exploration fonctionnelle respiratoire constitue une étape essentielle. La spirométrie permet, en plus de la mesure du VEMS, CVF et du VEMS/CVF, de mesurer le DEM 25%-75%. D'après plusieurs études c'est un marqueur de l'implication bronchique précoce chez les patients souffrant de rhinite allergique persistante [15, 16]. Chez nos patients, la spirométrie a mis en évidence une baisse des débits périphériques dans 282 cas alors que le VEMS était normal. La spirométrie permet, entre autres, d’évaluer la réversibilité du TVO aux bronchodilatateurs. Classiquement, ce test est positif chez les asthmatiques. Cependant, dans l'asthme sévère avec remodelage bronchique, cette réversibilité peut faire défaut [17]. Le taux de réversibilité rapporté dans notre série est de 55,1%. Ceci peut être expliqué par le fait que la spirométrie est souvent pratiquée chez des patients déjà sous traitement de fond (Corticothérapie inhalée ± β2 longue action). Adriel Gudiel H [18] a trouvé aussi un taux de réversibilité faible de 46,3%.

En présence d'une symptomatologie clinique atypique d'asthme, I'exploration fonctionnelle respiratoire prend également une valeur diagnostique considérable. En effet, elle permet de mettre en évidence une hyperréactivité bronchique (composante importante de la maladie asthmatique) par la réalisation de test de provocation bronchique non spécifique. Dans le cadre du bilan allergologique, les tests cutanés allergologiques constituent l’élément de base et l’étape essentielle. Ces sont des examens simples, de réalisation rapide, peu couteux et de grande sensibilité. La technique la plus couramment utilisée est celle du prick test [3]. Le dosage des IgE spécifiques permet aussi de confirmer l'origine allergique de l'asthme. Sa performance diagnostique est excellente avec des taux de sensibilité et spécificité de 85 à 95%. Ils sont parfois plus spécifiques mais moins sensibles que les prick-tests [3]. Ce dosage est réservé à des situations particulières: chez le nourrisson, vue la faible réactivité cutanée à cet âge, chez les sujets porteurs de dermatose étendue, chez les sujets ayant un dermographisme, chez les patients ayant un témoin positif inférieur à 2mm et en cas de discordance entre les données anamnestiques et les résultats des tests cutanés allergologiques [3]. Chez nos asthmatiques, le bilan allergologique a conclu à une prédominance de la sensibilisation aux acariens (91,2%), les pollens viennent en 2ème position (22,8%), on retrouve ensuite les phanères des animaux (12%), les blattes (4,9%) et les moisissures (3,7%). Dans la littérature, la prévalence de la sensibilisation à un allergène est variable d'une région à une autre et d'un pays à un autre. Dans notre étude, nous avons retrouvé un profil de sensibilisation relativement similaire à celui observé dans L'Europe du sud [19, 20] et les autres pays de l'Afrique du Nord ainsi que dans les autres séries tunisiennes avec une nette prédominance des acariens suivis de pollens [21–24]. En effet, ces pays partagent un climat et un environnement similaire (humide) favorisant le développement de ces types d'allergènes. Le diagnostic de sévérité constitue une étape déterminante dan la prise en charge de l'asthme. L'ancienne classification selon la sévérité faisait partie du bilan initial de l'asthme. Selon cette classification, seulement 1,9% de nos asthmatiques avaient un asthme sévère et la majorité (87,1%) avait un asthme intermittent et léger persistant. L'analyse des données de la littérature a objectivé les mêmes constatations à savoir que l'asthme allergique est souvent moins sévère que l'asthme non allergique [12]. La nouvelle classification de l'asthme selon le contrôle a permis d'optimiser la prise en charge et ainsi de déterminer un palier de traitement pour chaque patient. Pour nos patients, le suivi nous a fourni une appréciation sur le niveau de contrôle de l'asthme en se basant essentiellement sur les symptômes fonctionnels lors des dernières visites. Nous avons noté que 68,3% de nos patients étaient bien contrôlés, 24,8% partiellement contrôlés et 6,9% non contrôlés.

Une étude clinique épidémiologique allemande transversale portant sur 572 patients suivis en ambulatoire souffrant d'asthme allergique et qui étudie le contrôle de l'asthme évalué selon GINA, a montré que 65,4% des patients étaient bien contrôlés, 30,3% partiellement contrôlés et 4,4% non contrôlés [25]. Soit un résultat proche de celui retrouvé dans notre étude. Pour le traitement, la corticothérapie inhalée reste la base du traitement de fond de l'asthme persistant. Ce traitement permet de contrôler la maladie asthmatique dans la majorité des cas, de réduire les exacerbations de l'asthme, d'améliorer la fonction pulmonaire et de diminuer le recours au traitement de secours. Dans notre étude, la corticothérapie inhalée était prescrite pour la majorité de nos asthmatiques (67,6%). Il s'agit d'asthme persistant dans tous les cas. L'adjonction d'un β2 adrénergique de longue durée d'action à une corticothérapie inhalée a démontré son intérêt dans l'amélioration des symptômes ainsi que la fonction respiratoire et la prévention des exacerbations [26]. Dans notre étude, seuls 50 patients ont bénéficié d'un traitement par β2 adrénergique longue action. Ceci est essentiellement dû aux problèmes de prise en charge et au coût de ce traitement. La théophylline est une molécule bronchodilatatrice [27]. Elle peut être indiquée dans l'asthme en plus d'une corticothérapie inhalée lorsque cette dernière n'arrive pas à contrôler la maladie. Cependant, la théophylline reste moins efficace que les β2 adrénergiques de longue durée d'action avec des effets secondaires plus ou moins fréquents [28]. Dans notre étude, vu la non disponibilité des β2 adrénergiques longue action dans les structures hospitalières, la théophylline a été indiquée chez 204 patients. Le traitement étiologique prend une place importante dans la prise en charge thérapeutique de l'asthme allergique. Les mesures d’éviction ont démontré leur efficacité dans le contrôle de l'asthme dans la majorité des études. L'immunothérapie spécifique (ITS) est particulièrement efficace dans le traitement de la rhinite et la conjonctivite d'origine allergique [29]. Plusieurs études ont aussi conclu que l'immunothérapie est efficace dans le traitement de l'asthme, se traduisant par une diminution des symptômes, une baisse des besoins en médicaments, une réduction de l'hyperréactivité des voies aériennes, une réduction du risque de néosensibilisation chez les patients monosensibilisés et une réduction du risque d’évolution de la rhinite vers l'asthme [4, 29]. Dans notre série, l'immunothérapie spécifique a été réalisée chez 141 patients (12,5%) qui étaient monosensibilisés dans 83,6%. Il s'agissait essentiellement de désensibilisation aux acariens dans 98,5% des cas puis aux pollens. Cette ITS a été réalisée en cas d'asthme associé à une rhinite allergique dans 85,7% des cas.

## Conclusion

L'asthme est une maladie inflammatoire chronique des bronches, considérée comme la résultante de complexes interactions entre des facteurs génétiques et des facteurs environnementaux. Sa prévalence est en augmentation comme en attestent les multiples études épidémiologiques menées dans plusieurs pays, ce qui rend cette maladie un réel problème de santé publique. Les raisons de cette augmentation ne sont pas totalement connues, mais la charge allergénique accrue pourrait en être potentiellement responsable. L'asthme allergique est une affection répandue qui touche essentiellement le sujet jeune en pleine activité. Une prise en charge adéquate permet de contrôler la maladie et réduire ses répercussions non seulement sur le patient mais aussi sur la collectivité. Des mesures préventives agissant sur l'hygiène et sur l'environnement pourraient diminuer la fréquence de cette affection.
